# Why have nanotechnologies been underutilized in the global uprising against the coronavirus pandemic?

**DOI:** 10.2217/nnm-2020-0163

**Published:** 2020-05-28

**Authors:** Vuk Uskoković

**Affiliations:** ^1^Department of Mechanical & Aerospace Engineering, University of California Irvine, Engineering Gateway 4200, Irvine, CA 92697, USA

**Keywords:** COVID-19, infectious disease, interface, nanoparticle, nanotechnology, vaccine

## Abstract

Prior research on nanotechnologies in diagnostics, prevention and treatment of coronavirus infections is reviewed. Gold nanoparticles and semiconductor quantum dots in colorimetric and immunochromatographic assays, silica nanoparticles in a polymerase chain reaction and spike protein nanospheres as antigen carriers and adjuvants in vaccine formulations present notable examples in diagnostics and prevention, while uses of nanoparticles in coronavirus infection treatments have been merely sporadic. The current absence of antiviral therapeutics that specifically target human coronaviruses, including SARS-CoV-2, might be largely due to the underuse of nanotechnologies. Elucidating the interface between nanoparticles and coronaviruses is timely, but presents the only route to the rational design of precisely targeted therapeutics for coronavirus infections. Such a fundamental approach is also a viable prophylaxis against future pandemics of this type.

Classifiable as both animate and inanimate, viruses present a most curious type of physical systems known to humanity. In addition, the genetic makeups of humans and viruses are intimately related, given that nearly 10% of the human genome comprises endogenous retroviral DNA, while 30–40% of it comprises interspersed repetitive sequences that coordinate with the retrovirus genome components and affect the expression of neighboring genes [1]. The fact that a large percentage of human DNA is virally derived has raised questions over the extent to which viruses, their inanimateness notwithstanding, symbiotically co-evolve with higher forms of life through a horizontal gene transfer that they provide [2]. Aside from being responsible for crafting our genetic makeups to a finite extent, viruses are also responsible for a number of diseases striking humanity, some of which, like common cold, are relatively harmless, some of which, like SARS-CoV-2, are low- to high-risk, and some of which, like rabies or Ebola, entail bleak prognoses for the patients.

Coronaviruses are a subclass of viruses that infect the respiratory and/or gastrointestinal tracts of avian and mammalian species, with symptoms ranging from mild to serious. The structure, the pathology and the phylogeny of coronaviruses were the subjects of earlier research and review studies [3–5] and lie beyond the scope of this perspective article. Only a few basic remarks regarding the general structural features and properties of coronaviruses need be mentioned in this introductory section. First of all, coronaviruses are enveloped, nonsegmented, positive-sense RNA viruses; as such, their stability is lower, but the potential for mutation is higher compared with the DNA analogs. Because of the lack of the proofreading activity of RNA virus polymerases, the mutation propensity and the adaptive capacity of RNA viruses are comparatively high [6]. One consequence of this genetic variability is that a single RNA virus population normally exists in the form of an ensemble of mutant genotypes [7]. Morphologically, coronaviruses are spherical viruses and their name derives from the characteristic spikes dominating the particle surface, resembling a crown or the solar corona. This rather complex, helical symmetry nucleocapsid coating may be responsible for stabilizing the comparatively large RNA genome of these viruses, averaging at approximately 30 kb. In total, coronaviruses comprise four structural proteins, including spike (S; ∼150 kDa), membrane (M; 25–30 kDa), envelope (E; 8–12 kDa) and nucleocapsid (N; ∼45 kDa) ones, all of which could be used as targets for diagnostic assays, therapeutic actions or vaccine development schemes.

Out of a number of coronavirus species infecting birds and mammals, seven are pathogenic to humans. The most recent one, SARS-CoV-2 has caused a pandemic that spread all over the globe 3 months after the virus had first been reported in Wuhan, China. This situation has provided an occasion to test how promptly the scientific community can come together and reach a solution for the pandemic. This brief report arose from the will to join this concerted effort of scientists to curb the SARS-CoV-2 pandemic that has caused numerous deaths and hampered the daily life in many parts of the world as of early 2020. Aside from revisions carried out in early May 2020, it was fully written within 72 h in mid-March 2020, immediately following the declaration of the SARS-CoV-2 pandemic by the World Health Organization on 11 March 2020. At that point, it had been 3 months since the virus had first been reported in Wuhan, China, and yet it has gripped the globe, imposed an unprecedented pressure on the healthcare systems all the world over and quarantined billions of people on the daily basis. In a likely scenario, however, this pandemic, albeit catastrophic *per se*, may be but a drill and a prelude for its worse follow-up that may strike humanity in a not so far future, in which case a rapid and organized response of various disciplines within the scientific community shall be needed to find a quick solution for it and prevent the devastating loss of life. In that sense, this communication is concrete, but also conceptually experimental by being a part of the global test of the extent to which this response of the community will be such: concerted, to-the-point and capable of yielding a satisfactory solution in a relatively short period of time. The report presented here gives a succinct review and a personal point of view on the use of nanotechnologies in search of the solution for the current and the pending coronavirus pandemics. A particular emphasis is on highlighting and elaborating the lack of use of these technologies in spite of their proven potential to define new and improve the existing medical treatments. In general, such pointing at the gaps in the fabric of human knowledge is an essential step preceding their filling [8].

## Nanotechnologies: definition & literature search

Nanotechnologies are the term used to describe the application of material structures with the critical sizes under 100 nm [9,10]. Typically, materials composed of particles with at least one spatial dimension lower than 100 nm are classifiable as nanomaterials, while their application falls under the umbrella of nanotechnologies. At this point, the definitions become shaky and often break down, as many materials literally satisfy this criterion, but are hardly considerable as nanomaterials. One example are liposomes, whose sizes are not only right around the 100 nm limit that separates nanoscale objects from the microscale ones, but with their bilayer, vesicular structures and hollow cores they do not represent nanoparticles as authentically as ultrafine dispersions of inorganic structural entities do. Peptide and polypeptide monomers and oligomers with dimensions under 100 nm are structures that similarly satisfy the aforementioned definition, but are classifiable as nanostructures only under special conditions. These minor etymological issues aside, nanotechnologies have been used with success to increase the therapeutic effects of a number of agents with poor pharmacokinetics and pharmacodynamics [11–13]. As drug delivery carriers, they are effective in bringing the therapeutic agents closer to their target of action and/or crossing a number of biological barriers* en route* to the target from the site of administration, be it oral, parenteral or topical [14]. A myriad of chemicals effective in inhibiting diseases *in vitro* display poor inhibition rates *in vivo*, alongside causing systemic side effects, and the problem lies exactly in the lack of appropriate drug delivery technologies, the most prospective of which belong to the realm of nanotechnologies [15]. Here, it is especially high-risk patients who display precarious preconditions that benefit most from drug delivery technologies and that are, in turn, hit hardest by their absence. For, while healthy patients usually respond better to systemic pharmacotherapies utilizing no targeted carriers, to save lives of patients with preconditions, who turn out to be a particularly critical population for SARS-CoV-2 infections, cell and tissue targeting technologies may be a must. Also, with the diameter of coronaviruses averaging at approximately 125 nm [16,17] and the 20 nm long homotrimers of the spike protein protruding from their surface [18], the most natural physical structures to interact therewith may be expected to be of comparable sizes, in other words, 100 nm or under. This can be insinuated from studies demonstrating that the similarity in size of interacting entities maximizes the effects of their interaction, regardless of what constitutes the interaction in question [19].

**Figure 1.**
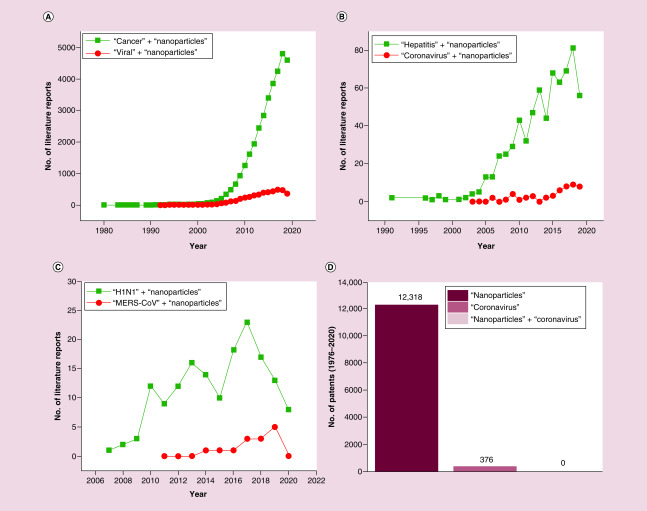
Bibliographic analysis. Total annual number of publications listed in the United States National Library of Medicine and containing the following keywords: ‘nanoparticles’ and either ‘cancer’ or ‘viral’; **(A)** ‘hepatitis’ or ‘coronavirus’; **(B)** ‘H1N1’ or ‘MERS-CoV’ **(C).** Search was performed at https://www.ncbi.nlm.nih.gov/pubmed/ on 14 March 2020. Total number of patents filed in the United States Patent Office and containing the word ‘nanoparticles’, the word ‘coronavirus’ or both of these words in the claims for the period 1976–2020 **(D).** Search was performed at https://patft.uspto.gov/netahtml/PTO/search-adv.htm using the ‘aclm/(keyword and keyword)’ operator on 15 March 2020. MERS-CoV: Middle East respiratory syndrome-related coronavirus.

In spite of these immense potentials, there is the impression that nanotechnologies have been underused in the research of ways to diagnose, prevent and treat the coronavirus infections. This is illustrated in Figure 1, which shows the annually published number of papers satisfying specific keywords and deposited in the National Library of Medicine (Figure 1A–C) or the total number of patents containing specific keywords in the claims (Figure 1D). First of all, the publications on nanoparticles in cancer have outnumbered those on nanoparticles in viral disease 7.0 ± 2.9-times annually on average since 2000 (Figure 1A). In contrast, fatality rates aside, more than 3600 viral species are currently known [20], which may infect more than 50% of the human population each year [21], whereas the annual cancer incidence rate is approximately 0.5% [22]. However, even within the sphere of nanotechnologies in viral diseases, coronaviruses have not been the prime focus of research interest. As it is shown in Figure 1B, hepatitis, as an exemplary viral disease, is a much more frequently studied subject than coronavirus in the context of nanotechnologies, with the annual number of publications on nanoparticles in hepatitis outnumbering those on nanoparticles and coronavirus 15.9 ± 11.3-times annually on average since 2010 (excluding 2013, when no publications on any coronavirus and nanoparticles were reported in the National Library of Medicine). Finally, nanoparticles have been more researched even in association with the H1N1 influenza infection than in association with the severest of seven human coronaviruses discovered so far, Middle East respiratory syndrome-related coronavirus (MERS-CoV), whose fatality rate was estimated at 35.6% (Figure 1C) [23]; the situation in the patent world is not significantly different is demonstrated by Figure 1D, which shows the number of patents filed in the United States patent office with the word ‘nanoparticles’ mentioned in the claims for all years to date equaling 12,318 and the number of patents with the word ‘coronavirus’ mentioned in the claims for the same period equaling 376. At the same time, a patent containing both of these keywords in the claims is yet to be filed.

## Review of nanotechnologies in coronavirus research

The use of nanotechnologies in the contest against the current and the future coronavirus pandemics will fall within three different categories: diagnostics, prevention and treatment. Studies focusing on one or more of these categories have already been reported and will be briefly reviewed in subsections that follow.

### Nanotechnologies in coronavirus diagnostics

Metallic, polymeric, silicon oxide, iron oxide and quantum dot nanoparticles have been used so far in coronavirus diagnostic assays. Among the metallic systems, the most commonly used by a large margin has been gold nanoparticles. These nanoparticles have most frequently been utilized for their characteristic surface plasmons that result in wavelength-selective absorption with relatively high molar extinction coefficients and the enhancement of the localized electromagnetic fields. One colorimetric assay, for example, made use of gold nanoparticles shielded by self-assembled double-layer DNA strands complementing the open reading frame 1a on the MERS-CoV E protein [24]. In a similar assay, the SARS-CoV E protein was immobilized on gold nanoparticles with the use of a gold-binding polypeptide as a mediator [25]. These gold nanoparticles were additionally functionalized with a green fluorescent protein and they displayed changes in absorbance and color upon the interaction with a complimentary antibody, thus allowing for the quantitative detection of SARS-CoV. Gold nanoparticles coupled to a monoclonal antibody were also used as a reagent for the detection of another coronavirus, porcine epidemic diarrhea virus (PEDV), in an immunochromatographic assay of swine stool samples [26]. They were also used as a coating over carbon electrodes immobilizing the recombinant spike protein S1 in a potentiometric immunosensor for the detection of MERS-CoV, with the role to increase the surface area, the electron transfer rate of the electrode and thus the cathodic peak current due to the reduction of the ferro/ferricyanide redox solution [27]. Gold nanoparticles with 20 nm in diameter were also used in a biosensor for the detection of SARS-CoV based on changes in the fluorescence of a fluorophore labeled with anti-SARS-CoV N protein secondary antibodies and bound to gold nanoparticles in response to the binding of the SARS-CoV N protein [28]. A colorimetric probe for the detection of MERS-CoV was also fabricated with the use of silver nanoparticles [29]. Functionalized with pyrrolidinyl peptide nucleic acid containing a positively charged lysine residue at the C-terminus, citrate-stabilized silver nanoparticles aggregated in the absence of complementary DNA, while in its presence they formed complexes between anionic DNA and cationic pyrrolidinyl peptide nucleic acid, which dispersed the nanoparticles and yielded a detectable color change.

Silica-coated superparamagnetic nanoparticles were utilized at multiple stages of polymerase chain reaction (PCR) assays for the detection of SARS-CoV genetic sequences [30]. Gold nanoparticles were also used in PCR assays for the detection of PEDV to increase the thermal conductivity of the genetic isolate mixture and thus allow for the target temperature to be reached faster, with reduced nonspecific amplification and increased specific amplification as a corollary [31]. Reportedly, the method displayed a 100-times higher sensitivity compared with the conventional PCR, with the detection limit of 2.7 × 10^-6^ ng/μl for PEDV RNA. The same type of nanoparticle-assisted assay was used to distinguish between the field and the vaccine strains of PEDV based on the deletion of the open reading frame 3 accessory gene in the genome of the latter strain [32]. In a more complex setting, gold nanoparticles were coated with oligonucleotides specific for transmissible gastroenteritis virus (TGEV) or PEDV, thus allowing for the magnification of weak signals from highly diluted serological or fecal samples using a PCR assay [33–35]. In general, nanoparticles implemented in PCR assays shorten the reaction time, enhance the signal amplification specificity and improve the detection sensitivity. Table 1 shows the comparison between the qualities of conventional PCR as the ‘gold’ standard for the detection of coronaviruses, including SARS-CoV-2, and of its nanoparticle-enhanced analog. As it is the case with the broader scopes of the interaction between nanoparticles and cells, the nanoparticle effects are pleiotropic, synergistic and not easily delineable from the mechanistic standpoint [36].

**Table 1.** Properties of conventional polymerase chain reaction as the ‘gold’ standard assay for the detection of coronaviruses compared with those of its nanoparticle-assisted analogs – nano polymerase chain reaction.

Property	Parameter	PCR	NanoPCR
Amplification efficiency	Doubling rate of DNA amplicons per cycle	90–105%	∼95%
Amplification efficiency	Threshold cycle	∼35	15–25
Analytical efficacy	Amplification time	∼2 h	30 min^-1^ h
Detection limit	Minimum detectable RNA copy number per reaction	∼1000	∼100
Analytical specificity	FP/(FP + TP)	15–35%	∼20%
Diagnostic sensitivity	TP/(TP + FN)	65–80%	80–100%
Precision	Repeatability coefficient of variation	1–10%	0.5–5%

The reader may consult the following references for more information: [37–42].

FN: False negative; FP: False positive; PCR: polymerase chain reaction; TP: True positive.

Photonic nanocrystals chemically functionalized with biomolecular probes have allowed for the detection of markers of viral disease through an antigen–antibody interaction, including those of SARS-CoV [43]. Semiconductor quantum dots conjugated with an RNA oligonucleotide were also shown to be sensitive to 422 amino acid residues long SARS-CoV N protein and capable of screening for the potential inhibitors of this protein in a biochip array [44]. ZnS-capped CdSe quantum dots labeled with a single-domain antibody targeting the membrane protein of PEDV were also used to visualize the viruses inside Vero cells [45]. Zirconium quantum dots were conjugated to anti-infectious bronchitis virus (IBV) antibodies, allowing the recognition and trapping of the complementary coronavirus antigen analyte and the subsequent concentration measurements by the addition of anti-IBV antibody-conjugated magnetoplasmonic nanoparticles separable in the external magnetic field [46]. One of the most complex diagnostic nanodevices for a coronavirus (PEDV) antibody detection designed to date is an electrochemiluminescent sensor utilizing two types of nanoparticles: ruthenium-doped silica to increase the signal amplification efficiency and minimize noise and nonspecific adsorption, and gold nanoparticle-modified graphene nanosheets as the substrate for antibody–antigen recognition to increase the specificity and sensitivity [47].

Overall, a substantial effort has been made to devise lateral flow strip tests for the detection of coronaviruses with the use of nanotechnologies, but converting a specific recognition observed in the laboratory setting into a clinical on-site device has proven challenging. One successful example comes from the immunochromatographic strip as a rapid, point-of-care test for IBV infection in chickens, employing monoclonal antibodies conjugated to gold nanoparticles and binding to the antigen of interest, after which the complexes are immobilized on the support matrix containing unlabeled antibodies [48]. Following the decision of the US FDA to permit clinically licensed labs to report on the results of in-house SARS-CoV-2 diagnostic assays without waiting for the Emergency Use Authorization approval on 28 February 2020, a lateral flow strip assay was devised, involving reverse transcription and isothermal amplification from RNA extracted from nasopharyngeal swabs of patients in the first stage, Cas12 detection of predefined coronavirus sequences in the second stage and the cleavage of a reporter molecule to confirm the detection of the virus in the third stage [49]. However, similar point-of-care assays based on the use of nanotechnologies and enabling the diagnosis of SARS-CoV-2 are still in the development stage in the private and the academic sectors [50].

### Nanotechnologies in the prevention of coronavirus infection

In the preventive category, nanoparticles have had a long history of use as adjuvants for the vaccine development against viral diseases in general. They are often able to promote a multifold increase in the antibodies titer in organisms immunized with nanoparticle-supplemented vaccines compared with the immune response provoked upon the treatment with nanoparticle-free formulations [51,52], with the caveat that their mechanism of action is often enigmatic. Although no clinically available vaccine has been made yet for any of the human coronaviruses, plenty of research has been conducted in this direction, some of which has relied on nanotechnologies. Various nanoparticle chemistries, including polypeptide, polymeric and metallic, have been used to achieve the immunostimulatory effect against coronaviruses.

**Figure 2.**
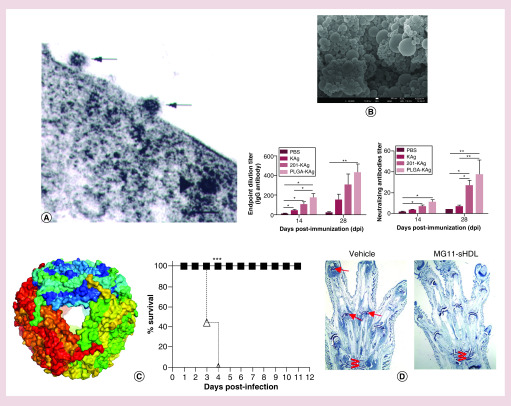
Nanotechnologies in coronavirus research. **(A)** Transmission electron micrograph of SARS-CoV viral particles entering a Vero E6 host cell by binding to the cell surface receptor (upper left arrow), then having their envelopes fuse with the cell membrane (central arrow) and nucleocapsids enter the cell (arrowhead). Bar is 100 nm. Reproduced with permission from [53], licensed with CC BY 3.0. **(B)** Poly(D,L-lactide-co-glycolide) nanoparticles loaded with inactive PEDV antigens (PLGA-KAg) increasing IgG and neutralizing antibody titers in sows relative to the titers in sows treated with saline and sows inoculated with the antigen alone (KAg and 201-KAg). Bar is 100 nm. Reproduced with permission from [68] © Elsevier (2017). **(C)** Schematic representation of a protein cage nanoparticle showing individual protein subunits and the survival of mice infected with SARS-CoV after the treatment with saline (empty triangles) or with the protein cage nanoparticles (black squares). Reproduced with permission from [83], licensed with CC BY 3.0. **(D)** Toluidine blue staining of the fore paws of the vehicle control mice showing moderate inflammation and cartilage damage with moderate pannus and bone resorption in all the joints and of mice treated with the SARS-CoV-derived peptide MWKTPTLKYFG (MG11) delivered with spherical high-density lipopeptide nanoparticles, showing no inflammation and minimal cartilage damage. Arrows identify affected joints. Tipped W denotes the wrist. Reproduced with permission from [112], licensed with CC BY 4.0. PBS: Phosphate-buffered saline; PEDV: Porcine epidemic diarrhea virus; PLGA: Poly(D,L-lactide-co-glycolide); sHDL: Spherical high-density lipopeptide nanoparticles.

The trimeric S glycoprotein mediates the attachment of the virus to the host receptor (Figure 2A) [53] and presents the most logical target on the coronavirus particle. The least complex nanoparticles reported for these ends for coronaviruses such as SARS-CoV and MERS-CoV have been those formed from the direct recombinant spike protein as an antigen and administered with adjuvants such as alum and/or Matrix M1 [54–56]. The current trend in immunization studies, however, is to utilize selected sequences of the spike protein, given that the use of the full-length protein, albeit eliciting the protective immune response, has also enhanced the antibody-dependent viral entry into human B cells [57], the virulence of hepatitis [58] and pulmonary eosinophilia [59]. Conjugation of the full-length S protein of SARS-CoV to gold nanoparticles has not mitigated the allergic inflammatory response nor reduced the eosinophilic infiltration [60]. Correspondingly, self-assembled recombinant oligopeptide nanoparticles with the amino acid sequence corresponding to residues 1156–1178 on the SARS-CoV spike protein promoted the neutralization activity of antisera in an *in vitro* infection inhibition assay without any adjuvants [61]. Also, the expression of only the B-cell epitope derived from the second heptad repeat (HR2) region of the IBV spike protein co-displayed with flagellin yielded nanoparticles for the immunization of poultry [62]. The S protein of MERS-CoV without its transmembrane and cytoplasmic domains assembled into nanoparticles was proposed as another candidate for a vaccine against MERS-CoV [63]. One step ahead of this approach would be to go beyond the simple spherical nanostructures and create more complex morphological symmetries using tertiary structural elements of coronavirus proteins as building blocks. Such structures have been designed *in silico* [64,65], but their physical assembly is a challenge. Nevertheless, there are notable examples, one of which is the use of RNA as a chaperone and protein-folding vehicle that directs the folding and the assembly of recombinant monomeric vaccine antigens containing the receptor-binding domain of MERS-CoV in bacterial host cells into complex nanoparticle geometries with improved immunological functions [66].

As for polymeric nanoparticles as adjuvants and/or antigen carriers, polyethylene nanoparticles were used to deliver SARS-CoV pDNA encoding for the spike protein and thus immunize mice via an intranasal route of administration, with a higher S-specific IgG1 concentration in the sera and a higher secretory IgA concentration in the lung wash than those in mice treated with the DNA alone, without the nanoparticle carrier [67]. An intranasal inoculation with poly(D,L-lactide-co-glycolide) (PLGA) nanoparticles loaded with denatured PEDV antigens similarly led to enhanced IgG and IgA antibody titers in pregnant sows immunized with the antigen-loaded nanoparticles relative to the titers in sows inoculated with the antigen alone (Figure 2B) [68]. Chitosan nanoparticles were used to entrap an inactivated antigen for avian IBV and they produced a markedly mucosal immune response and provided protection against the infection at both local and systemic sites after an oculo-nasal administration to chickens [69]. Biotinylated chitosan nanoparticles were functionalized with a fusion protein vector to achieve the selective targeting of dendritic cells and deliver the SARS-CoV N protein pDNA to them, leading to an enhanced mucosal IgA concentration and an enhanced systemic presence of IgG against the N protein following the intranasal administration [70]. N,O-carboxymethyl chitosan is another chitosan derivative that was used as both adjuvant and delivery carrier for coronavirus vaccine antigens [71]. Thanks to the abundance of constitutive amine groups, chitosan is a positively charged polymer relying on a favorable electrostatic attraction to adhere to and permeate epithelial monolayers and cell membranes and achieve the intracellular delivery of the genetic load [72,73]. Outside the vaccine domain, but still within the preventive area, *N*-(2-hydroxypropyl)-3-trimethyl chitosan nanoparticles cross-linked with genipin were used to adsorb coronaviruses NL63 and OC43 and were proposed as effective for the purification of waters contaminated with pathogenic coronaviruses [74].

As far as inorganic nanoparticles are concerned, gold nanoparticles were used to deliver a TGEV antigen and immunize animals by those means, leading to an enhanced respiratory activity of peritoneal macrophages and higher plasma IFN-γ levels compared with those achieved by the delivery of the attenuated virus alone [75]. In another study, higher levels of IFN-γ, IL-1β and IL-6 were measured in animals immunized with the similar gold nanoparticle–TGEV antigen complexes than those in intact animals or animals challenged with the antigen alone [76]. Vaccination with gold nanoparticles functionalized with the S protein as an antigen also produced stronger antibody titers, enhanced the lymphatic antigen delivery and increased the splenic T-cell response, while reducing the infection symptoms in an avian model of coronavirus infection compared with the vaccination with a commercial whole inactivated virus vaccine [77]. This succinct catalog of nanotechnologies used in the prevention of coronavirus infection does not include more universal nanoproducts of relevance for the ongoing pandemic, such as nanofiber-based protection masks or nanoemulsion-based hand sanitizers.

### Nanotechnologies in the treatment of coronavirus infection

As far as the treatment of organisms infected with a coronavirus is concerned, various strategies for mitigating the ongoing infection have been explored with the use of nanotechnologies, but to a significantly lesser extent than for diagnostic and preventive purposes. Silver nanoparticles epitomize a nanomaterial with antimicrobial properties and although they have been exploited to date mostly against various bacterial and fungal species, their activity against viruses such as HIV, hepatitis viruses and influenza viruses has been noted too [78]. Therefore, it should not be surprising that silver nanoparticles of various morphologies, including 20 nm-sized spheres and nanorods with 10 nm in diameter and the aspect ratios of 6:1 and 40:1, were effective in diminishing the infectivity of TGEV in swine cells [79]. However, although these nanoparticles could act as a virucidal agent to isolated viruses and inhibit the viral entry into the cell, it is uncertain whether they can inhibit and/or destroy the intracellular nucleocapsids and rescue the infected cell. It is likely that such finer targeting strategies would require the nanoparticles to be conjugated to specific ligands for molecular recognition of viral nucleotide or protein sequences, so that the latter can be selectively inhibited inside the host cell.

Graphene oxide comes from a family of 2D, monoatomically thin nanomaterials and it showed an intrinsic antiviral activity against PEDV among other viruses, significantly higher than that of its precursor, graphite oxide [80]. However, a study comparing the antiviral activity of pure and silver nanoparticle supplemented graphene oxide with respect to feline coronavirus resulted in 25% of infection inhibition by the graphene/gold nanohybrid and only a 16% inhibition by graphene alone [81]. Interestingly, the activity of both nanomaterials was finite against the representative coronavirus and nil against an envelope-free infectious bursal disease virus, suggesting that the disruption of the outer layers of the viral particle is the most likely mechanism of action of these nanoparticles.

Organic nanoparticles have also been investigated for the treatment of coronavirus infections. For example, poly(ethylene glycol)-block-poly(lactide-co-glycolide) nanoparticles were used with success to deliver a vacuolar ATPase blocker, diphyllin, and prevent the endosomal acidification-dependent cytoplasmic entry of feline coronavirus [82]. Also, cage-like heat shock protein nanoparticles were designed to accelerate the clearance of respiratory viruses after the primary infection, but also elicit an immunoprophylactic response in the lung that protected the host against SARS-CoV (Figure 2C) [83].

When it comes to devising appropriate therapies for SARS-CoV infections, combining insights from the basic cell and molecular biology with antiviral nanotechnologies may be the most prospective route worth taking. For example, the treatment strategies currently employed in the pulmonary clinics all revolve around the use of anti-inflammatories in the attempt to downstream the production of cytokines and ‘put out the fire’. With the use of the targeting approach enabled by nanoparticles, however, a more effective and ‘cooler’ set of therapeutic strategies could be conceived, revolving around upregulating the production of endogenous protective factors identified through basic molecular biology technologies. One of them may be the LIF, which serves as a stem cell growth factor whose production decreases with age. Triggered by the binding of the virus to type II epithelial cells in the alveolar wall, the production of LIF facilitates the viral clearance and prevents the vascular leak, thus protecting the alveolar tissue in the lung from collateral inflammatory damage and promoting regenerative repair [84]. The PLGA nanoparticles were used earlier with success for the targeted delivery of LIF in the treatment of multiple sclerosis [85,86] and similar strategies hold the potential for treating SARS infections with a greater selectivity and efficacy than the conventional therapies. Another viable set of therapeutic approaches may be based on the targeted delivery of inhibitors of TMPRSS2 serine protease required for the initiation of the S protein of SARS-CoV after its binding to the ACE2 receptor on the alveolar epithelial cells of the lung [87,88].

## Toward understanding the nanoparticle–virion interface

 Most nanoparticles used in the treatment of bacterial diseases owe their mechanism of action partially or wholly to the ability to disrupt the bacterial cell wall. Such is the case with silver [89], gold [90], copper [91] and zinc oxide [92] nanoparticles. Even nanoparticles whose target lies internally, inside the bacterial cell, must cross the membrane in a process that is not natural as in the case of eukaryotic cells, which are, unlike prokaryotes [93], equipped with the machinery for endocytosis. Likewise, poking through the viroid envelope is likely to be the first action performed by an antiviral nanoparticle in contact with the virus. This process leads to an increased hydrophilicity of the envelope and is often sufficient to attenuate the viral particle [94]. To a large extent, therefore, the utilization of nanotechnologies for the prevention and treatment of infections caused by coronaviruses is an interfacial issue. If the interface between nanoparticles on one side and virus nucleocapsid shells and envelope glycoproteins on the other was understood better, important steps in the direction of the design of nanoparticles with selective effects – lethal to viral entities and harmless to the host cells – would be made.

However, to be effective, the interaction of nanoparticles and viral entities cannot end with the viroid coating disruption after the infection of an eukaryotic cell has occurred and the viral nucleic acid has undergone replication in the host cell. In those cases, different targeting strategies are needed to halt the replication and silence the replicating code. Current strategies for achieving so in therapies for coronavirus infections are largely indirect, leading to broad variations in the outcome across the population. The most commonly used antiviral drugs in such therapies, including the interferons ribavirin, remdesivir and others, count among such indirect solutions. One avenue where nanotechnologies could improve this approach is by making it more direct thanks to the targeting effect facilitated by the nanoparticles. This strategy may not always give the desired results, as it was the case with the delivery of ribavirin by lecithin-containing liposomes, which improved neither the survival rate nor severity of disease in kittens infected with feline infectious peritonitis virus and treated with free ribavirin or saline [95]. However, there have been cases when the delivery of the same antiviral drug, ribavirin, with gold nanoparticles [96] or lactosaminated poly-*L*-lysine [97] augmented the antiviral activity of the drug against measles and hepatitis C, respectively. Here, the comparatively large RNA genome (∼30 kb) of coronaviruses [98] can be considered a facilitator of the design of direct RNAi technologies. Different RNA delivery strategies, including the delivery of miRNA or siRNA, which partially or completely pair with the target RNA [99], respectively, with the use of nanoparticle vehicles have been considered for a number of viral diseases, including HIV [100] and hepatitis [101]. siRNA and miRNA sequences usable as antiviral therapeutics against MERS-CoV and other human coronavirus infections have been computationally predicted [102] and nanoparticle-based strategies for their delivery discussed along with the critical challenges [103], including immunogenicity, the enzymatic degradation in the lysosome and elsewhere, the inefficient cellular uptake, undesired effects at distant sites, clearance through kidney filtration and nonspecific uptake by the reticuloendothelial system. However, the real progress in the technologies for targeting intracellular viral genetic codes is yet to be made.

To achieve the aforementioned selective targeting effects, bare nanoparticles will likely prove insufficient and their hybridization with specific ligands will be necessary. Here, however, optimizations are critical to ensure that the interaction between the core nanoparticle and the therapeutic load is optimal and neither too strong nor too weak. Lateral interactions between ligands and solvent interactions complicate things here, especially when the nanoparticles are multicomponent and the solvent complex in composition and variable across different physiological media, including cytoplasm, the extracellular space, blood, gut and so on. Water, itself, is a reactive solvent that can significantly alter the interaction between the adsorbate and the substrate through hydration effects, electronic structure modification or proton channeling, but when hydrodynamic effects, structuration due to spatial confinement and the diverse chemical makeup of biological media are added to the picture, the fate of the complex becomes rather uncertain and unpredictable. One example of the optimal interaction is that of calcium phosphate nanoparticles and the genetic load, which gets released only when the nanoparticles begin to dissolve in the late endosome thanks to their unique pH-dependent solubility profile [104]. An example of a nonoptimal interaction is that of drugs covalently bound to gold nanoparticles, which often undergo endosomal entrapment upon entering the cell [105], thus threatening the degradation of the therapeutic cargo before it has reached the target, in part because of the excessively strong bond between the drug and the carrier. One curiosity here is that nanoparticles in a biological milieu spontaneously form a protein corona around them, which largely modifies the properties of bare nanoparticles in these milieus [106]. A simple endocytosis or exocytosis of a nanoparticle is sufficient to fundamentally change its surface composition [107] and with it the surface–drug–solvent and nanoparticle–virus/cell interactions. Moreover, it has been largely uninvestigated whether a similar corona forms around viral entities traversing the biological tissues and if it does, to what extent it alters their fate in various tissues. It is, however, known that this protein corona forming around viruses inside the organism affects the viral infectivity and the immune cell activation [108]. Therefore, a thorough investigation of the affinity of nanoparticles for viral particles would have to include the effects of this additional corona into analysis. Additionally, computational studies on the conformational changes and the stability of the envelope and the spike protein of coronaviruses upon the contact with nanoparticles with different chemical compositions, curvatures, topologies and terminations will be essential in this design of nanoparticles for the intracellular attenuation of viruses, after the infection has occurred.

All in all, systematic basic studies on the interface between nanoparticles and coronaviruses in extracellular and intracellular environments take time, but present the only route to the rational design of precisely targeted therapeutics for coronavirus infections. Nevertheless, an overarching impression is that all that has been done so far in terms of understanding this fundamental interface between nanoparticles and coronaviruses, albeit indisputably meaningful, is only the tip of an iceberg. Studies probing the direct interface between the viral particles and nanoparticles of various chemistries and physical characteristics – for example, morphology, size, surface charge, faceting and so on – at the atomic and nano-scales will prove to be of critical importance for setting up the base for the rational design of advanced, nanotechnology-assisted modes of therapy for coronavirus infections. Simultaneously, this fundamental knowledge will prove to be useful in allowing the conversion of various viral entry and immune evasion strategies into therapeutic strategies. Such examples abound in the literature with respect to viruses in general and they include the isolation and reassembly of the viral matrix proteins and surface glycoproteins into nanoparticle shells capable of promoting an effective encapsidation and delivery of therapeutic nucleic acids or proteins to cells of the recipient organisms [109,110]. Insights into the coronavirus structures have already yielded impetuses in this direction. For example, the expression and assembly of the native structural proteins of NL63 coronavirus in the form of a nanoparticle yielded vectors for the delivery and transduction of genes inside the ciliated cells of the respiratory tract [111]. The molecular mechanism by which SARS-CoV modulates the host immune response was also the basis for the design of a SARS-CoV fusion peptide sequence as an immunomodulatory peptide targeting the T-cell receptor and protecting against bone and cartilage damage after being delivered by lipopeptide nanoparticles mimicking the native human high-density lipoproteins (Figure 2D) [112].

## Conclusion

Despite decades of research, no antiviral therapeutics that target human coronaviruses have been designed. The absence of reliable options for inexpensive and fast diagnosis or for the prevention and treatment of SARS-CoV-2 and most other coronavirus infections in humans may largely have to do with the insufficient or inadequate research on nanotechnologies in viral disease. It is deemed that with a greater proficiency in regard to the interaction between nanosystems and viruses, the progress in hampering the spread of SARS-CoV-2 may have been more technologically advanced and less meagre in effect. This perspective article has been both a constructive critique and motivation for deeper understanding of the interactions between nanoparticles of various chemistries and physical characteristics on one side and coronaviruses on the other at the most fundamental levels. Insights into such fundamental interactions may not bring about an immediate practical benefit, but they will prove essential in the arduous and long-term process of rationally designing precise and effective therapeutic strategies, the development of which is invariably rooted in the knowledge of basic scientific phenomena.

However, until sufficiently rich fundamental insights are gathered and conditions for the rational design of therapeutic formulations gained, trial-and-error will, sadly, continue to be the prime mode of discovery of therapies for SARS-CoV-2 and other coronavirus infections. One confirmation of this model has come from the unexpected endorsement of the immunomodulator sarilumab (Kevzara), the monoclonal antibody approved by the FDA for the treatment of rheumatoid arthritis, as effective against pneumonia caused by SARS-CoV-2 and in use with reported success in the Italian hospitals as of March 2020 [113]. In fact, to conclude, the story of science, especially the pharmaceutical, is similar to that behind the Bor mine in eastern Serbia. Believed to conceal large reserves of gold, it was exploited as far back in time as in the Roman Empire [114], but only copper was found in it, accidentally, at the beginning of the 20th century [115]. Today, the mine is one of the world’s largest reserves of copper, an element equally significant for modern technologies as gold. Likewise, in search of the solution for the ongoing coronavirus pandemic, it may happen that we come across interfaces that would benefit some other, more general processes in virology and broader realms of medical science.

One of such insights toward which the findings pertaining to the infection by SARS-CoV-2 implicitly point is its synergy with other viral, bacterial or even fungal pathogens. In this author’s opinion, if recognized and systematically scrutinized, this insight may lead to a similar paradigm shift in virology as that experienced by bacteriology in the last decade with the introduction of the concept of the microbiome. That knocking down a harmless, noninvasive pathogen from the population of a microbiome can trigger the proliferation of its invasive member and lead to the onset of the disease is a widely accepted model in bacteriology today, but similar concepts are yet to be proposed and tested in the domain of virology, and yet this all may change due to insights gained during the effort to untangle the symptomatic from the asymptomatic pathology of SARS-CoV-2. With the transition to the microbiome paradigm, the language of bacteriology has had to fundamentally change, embracing more statistical and systemic frameworks, and the same may happen to the field of virology thanks to research on SARS-CoV-2, with a hopefully lifesaving impact on the treatment and prevention of a number of other viral infections. At this point, we may recall once again the golden age that began with the discovery of copper, not gold, in the mines of Bor, while still cautiously remembering the images of its dilapidation at the end of the 20th century due to the country’s economic collapse. For, the radical focus of researchers and the community on mitigating the SARS-CoV-2 pandemic may bring about numerous benefits for the healthcare and beyond just as well as it may make humanity vulnerable to other public health risks as well as socioeconomic issues and geopolitical instabilities that threaten its welfare from more inconspicuous and clandestine of angles.

On a more optimistic note, a pandemic like the SARS-CoV-2, the tragic loss of life it has caused notwithstanding, can serve as a wakeup call and an opportunity for humanity to reassess its preparedness for another, possibly worse pending pandemic, viral or bacterial, and timely devise more holistic and cooperative approaches across sciences and other disciplines, alongside races and nations, to cope with it. There is a hope that in this process, as during the unfolding of many other similarly adverse events in history, humanity will come together, reach a higher level of solidarity and allow many of the artificial boundaries drawn across its face to be erased by the empathy and the selfless allegiance to the good of humanity and our neighbors. Perhaps, in such course of events, a virus like SARS-CoV-2 may not destroy or sever us, but help us heal.

## Future perspective

The ongoing SARS-CoV-2 pandemic has been met with an unprecedentedly severe response of humanity in the effort to curb it, with little to moderate success so far. The interest of researchers in SARS-CoV-2 has exploded since the pandemic was declared on 11 March 2020, transforming coronaviruses from one of the least attractive subjects in medicine to the hottest practically overnight and demonstrating the volatility of trends in research, especially medical. New findings are reported daily, as more and more researchers from a variety of disciplines are being recruited to provide an empirical or intellectual input to this global effort, most often with no direct financial incentives, but with purely altruistic goals in mind. This sheds hope that the humanitarian face of medical research and practice might prevail in the long run over everything in them that is tangled in devious political webs and driven by financial or other self-interests. SARS-CoV-2 could also mark the beginning of a similarly concerted assault on infectious diseases *per se*, including the multidrug-resistant bacterial ones, whose pending pandemic has been yet another time bomb that humanity is sitting on. There is little doubt that nanotechnologies will play a major role in this upcoming wrestles against the invasive microbes that threaten our survival. Still, as with every offensive, vulnerabilities are being revealed all over, and it is hoped that science and humanity will learn how to patch them up before they take their toll and cause irreversible impairments to the physical and the mental health of us and, even more importantly, of generations that are blooming and are yet to emerge in full stature on this stage.

**Executive summary**

**SARS-CoV-2 pandemic**

- SARS-CoV-2 pandemic was declared by the World Health Organisation on 11 March 2020.
- Research interest in coronaviruses soared with the outbreak of SARS-CoV-2.
- The outbreak of SARS-CoV-2 has provided an occasion to test how promptly the scientific community can come together and reach a solution for the ongoing pandemic.
- The pandemic has shed light on the lack of fundamental scientific knowledge utilizable in the prevention and treatment of viral infections.

**Underuse of nanotechnologies**

- The absence of antiviral therapeutics that target SARS-CoV-2 might be largely due to the underuse of nanotechnologies in virology.
- There is a disparity between the underuse of nanotechnologies in the effort to halt the SARS-CoV-2 pandemic and their proven potential to define new and improve the existing treatments in medicine.
- Nanoparticles can enhance the performance of diagnostic assays, serve as adjuvants in vaccine formulations and promote targeted delivery of antiviral therapeutics.

**Prior research on nanotechnologies & coronaviruses**

- Review of the prior use of nanotechnologies in coronavirus research is divided into three categories: diagnostics, prevention and treatment.
- The use of nanoparticles in diagnostics and prevention of coronavirus infections has been notable, while their use in devising new or amending the existing treatments has been only sporadic.
- Nanoparticles of gold and silica have improved the analytical efficacy, specificity, sensitivity and precision of polymerase chain reaction as the current ‘gold’ standard for the detection of coronaviruses, including SARS-CoV-2.
- Nanoparticles of the coronavirus spike protein sequences, of gold and of polymers such as polyethylene, poly(D,L-lactide-co-glycolide) or chitosan have been used with success as adjuvants in vaccines protecting against coronavirus infections.
- Nanoparticles noted for their antibacterial activities, such as silver and gold, have been used as virucidal agents to neutralize the infectious viral particles *in vitro* and *in vivo*.
- Rather than suppressing the cytokine storm, the use of the targeting approach enabled by nanoparticles can lead to therapeutic strategies aimed at upregulating the production of endogenous protective factors identified through basic molecular biology technologies.

**Interface between nanoparticles & coronaviruses**

- Studies probing the interface between viruses and nanoparticles at the atomic and nano scales are required to set up the base for the rational design of advanced, nanotechnology-assisted modes of therapy for coronavirus infections.
- In search of the solution for the ongoing coronavirus pandemic, interfaces of benefit for other, more general processes in virology and broader realms of medical science may be discovered.
